# A Qualitative Analysis of Self-Harm and Suicide in Sri Lankan Printed Newspapers

**DOI:** 10.1027/0227-5910/a000687

**Published:** 2020-05-05

**Authors:** Jane Brandt Sørensen, Melissa Pearson, Gregory Armstrong, Martin Wolf Andersen, Manjula Weerasinghe, Keith Hawton, Flemming Konradsen

**Affiliations:** ^1^ Department of Public Health, University of Copenhagen, Denmark; ^2^ School of Clinical Sciences and Community Health, The University of Edinburgh, UK; ^3^ Nossal Institute for Global Health, Melbourne School of Population and Global Health, University of Melbourne, Australia; ^4^ Faculty of Medicine and Allied Sciences, Rajarata University of Sri Lanka, Sri Lanka; ^5^ Centre for Suicide Research, University of Oxford, UK

**Keywords:** self-harm, suicide, media, Sri Lanka, thematic analysis

## Abstract

**Abstract.** *Background:* Media reporting may influence suicidal behavior. In-depth exploration of how self-harm and suicide are portrayed in newspaper articles in a middle-income country such as Sri Lanka is lacking. *Aims:* We aimed to explore how self-harm and suicide are portrayed in Sri Lankan printed newspapers. *Method:* Seven English- and Sinhala-language Sri Lankan newspapers were screened for articles reporting on self-harm and suicide (December 1, 2014 to January 31, 2015). A thematic analysis was conducted. *Results:* In the 78 articles identified for analysis, certain aspects were overemphasized (inappropriate behavior) and others underemphasized (alcohol and complexities of self-harm). Explanations of self-harm were one-sided and a suicide prevention narrative was lacking. *Limitations:* Another time-frame and inclusion of Tamil newspapers as well as social media and online publications would provide additional understanding. *Conclusion:* The study found an indication of simplistic reporting. Greater focus on prevention and a nuanced portrayal of self-harm could reduce stigma and imitative behavior.

Self-harm and suicide constitute a significant global public health problem (World Health Organization [WHO], 2014). In this study we define *suicide* as "the act of deliberately killing oneself," whereas *self-harm* captures self-injury or self-poisoning regardless of motivation and intent (Hawton & van Heeringen, 2000). Responsible media reporting is an important self-harm and suicide preventive measure (Bohanna & Wang, 2012). Specifically, there is a relationship between media reporting practices in covering suicide and subsequent suicidal behavior in vulnerable individuals – especially if they can identify with the person who died or when the suicide seemed to solve problems (Pirkis, Mok, Robinson, & Nordentoft, 2016). Suicide reporting can also have a preventive effect when stories focus on suicidal ideation not followed by self-harm (Niederkrotenthaler et al., 2010). Further, responsible reporting can de-stigmatize and educate about suicide, provide information about help-seeking, and may lead to policy interventions (Bohanna & Wang, 2012; WHO, 2017). 

The WHO region of South-East Asia includes countries with the highest suicide rates globally. Almost 40% of suicides occur there (WHO, 2014). In Sri Lanka, high suicide rates peaked in the 1990s and have since declined (Knipe, Chang, et al., 2017). However, self-harm and suicide remain significant public health problems (Knipe, Gunnell, & Eddleston, 2017). To counter this, few resources guiding the media on appropriate reporting have been established. The *Suicide Sensitive Journalism Handbook* contained an analysis of 84 newspaper articles and recommendations for Sri Lankan media professionals to engage in sensitive reporting (Deshapriya, Hattotuwa, & Jempson, 2003). Furthermore, some guidance on media reporting of suicide was provided in the Editors' Guild of Sri Lanka's *Code of Professional Practice* (The Editors' Guild of Sri Lanka, 2014). 

Our recent publication examining the quality of reporting on episodes of self-harm and suicide in Sri Lankan printed newspapers found that compliance with recommendations was limited (Sørensen et al., 2018) in regard to reporting means of self-harm in the headline (53%), detailing individual characteristics (100%), using insensitive language (58% of English articles), and attributing one single cause to self-harm (52%). No information about help-seeking was included in the articles (Sørensen et al., 2018). 

While the connection between the media and suicide has been explored in international peer-reviewed literature, it has rarely been done in-depth in a low- or middle-income setting. The aim of this study was to explore how self-harm and suicide are portrayed in Sri Lankan Sinhala- and English-language printed newspapers over a 2-month period during 2014–2015. 

## Method 

The included newspaper articles were selected from three Sinhala (*Lankadeepa, Divaina, Ada*) and four English (*Daily News, Daily Mirror, The Island, Ceylon Today*) national, printed, broadsheet newspapers, chosen on the basis of popularity and circulation (Table 1). The main languages spoken in Sri Lanka are: the two official and national languages Sinhala (spoken by approximately 87%) and Tamil (29%) as well as English, which is primarily used in scientific and commercial urban areas (10%; Central Intelligence Agency, 2019). 

**Table 1:** Newspaper language, circulation, and characteristics

Newspapers	Publisher	Language	Circulation	Ownership
Lankadeepa	Wijeya Newspapers	Sinhala	250,000 (Daily Lankadeepa)	Private
			560,000 (Sunday Lankadeepa)	
Divaina	Upali Newspapers	Sinhala	156,000 (Daily Divaina)	Private
			340,000 (Sunday Divaina)	
Ada	Wijeya Newspapers	Sinhala	110,000	Private
Daily News	Associated Newspapers of Ceylon Limited	English	88,000 (Daily News)	State
			175,000 (Sunday Observer)	
Daily Mirror	Wijeya Newspapers	English	76,000 (Daily Mirror)	Private
			330,000 (The Sunday Times)	
The Island	Upali Newspapers	English	70,000 (Daily Island)	Private
			103,000 (Sunday island)	

For a 2-month period (December 1, 2014 to January 31, 2015) the newspapers were searched for articles reporting on self-harm and suicide. This time was chosen owing to the availability of staff resources. Articles and commentaries were included when they reported on self-harm and/or suicide and excluded when: self-harm or suicide was not mentioned; the theme was suicide bombing; the text focused on children; and when content was fictional. MW hand-searched the Sinhala newspapers and MWA the English ones for reports of self-harm and suicide. Sinhala articles were translated into English by an English literature graduate student. Individuals, whose cases of self-harm were portrayed in newspapers are presented with pseudonyms to safeguard anonymity.

### Data Analysis

An inductive, thematic analysis was conducted to identify, analyze, and report common themes on self-harm and suicide within the data (Braun & Clarke, 2006). Articles were uploaded to NVivo11 and carefully read and re-read. After initial coding by the first author (JBS), themes were discussed within the research team, made up of social scientists, health and mental health researchers from Sri Lanka, Denmark, Australia, and the United Kingdom. All possess significant experience within the suicide research field. Two parts of the analysis were inspired by critical discourse analysis (CDA): *intertextuality* or emerging discourses across articles, where the developments in the same story are portrayed over time, and *themes not included in the texts*, since meaning is communicated as much by what is excluded as included (Richardson, 2007). 

## Results 

### Newspaper Article Characteristics

**Figure 1:**
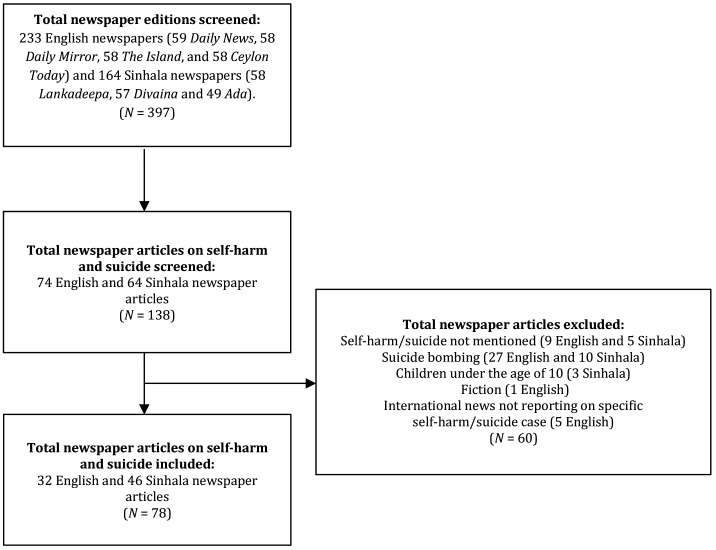
Newspaper article selection process.

The selection process is described in Figure 1. In total, 164 Sinhala and 233 English newspaper editions were screened for articles reporting on self-harm or suicide. Of these, 138 articles referred to self-harm or suicide; 60 of these articles did not mention *self-harm*, *suicide*, or referred to *suicide-bombing* and were thus excluded. In total, 78 articles were included in the study (46 Sinhala and 32 English), primarily from the Sinhala-language newspapers *Divaina* and *Lankadeepa* and the English-language newspaper *Ceylon Today*. Of all articles, 73 reported on specific episodes of self-harm/suicide (44 male and 29 female cases): 47% reported on suicide deaths; 17% on nonfatal self-harm; 18% debated whether it was self-harm, crime, or an accident; and 4% reported about nonfatal self-harm and suicide in the same report. One article reported on suicidal ideation. Five articles did not report on a specific case of self-harm or suicide. Six articles reported on suicides outside of Sri Lanka. 

Table 2 provides an overview of the different categories of newspaper articles. Of the 73 newspaper articles, 39 were brief crime reports published in English- and Sinhala-language newspapers providing personal characteristics such as name, age, area of residence, and method of self-harm. Self-harm/suicide episodes were portrayed in depth in 29 of the 73 newspaper articles, following a basic narrative structure: introducing individuals and setting (the individual who self-harmed and close relations), introduction of complications (troubles endured by the individual who self-harmed), and climax where complications are overcome (the case of self-harm) (Richardson, 2007). Of these 29 feature articles, eight were written in a short-story style including perceived emotions and conversations, seemingly covering actual cases of self-harm/suicide. Health education and information about suicide were provided in six commentaries, four of which were published in the English-language newspaper *Ceylon Today*. Four of the commentaries focused on explanations of self-harm/suicide, emphasizing mental illness, social and cultural factors; one focused on means of self-harm and another educated about how suicide is not a legal defense in Sri Lanka. All articles reporting on self-harm/suicide in Sri Lanka referred to Sinhala individuals except for two articles describing self-harm by a Tamil individual. 

**Table 2:** Overview of the different categories of newspaper articles included in the study

	Sinhala	English	Total
Crime reports	23	16	39
Feature article	18^a^	11	29
Commentary	2^a^	5	7
Court accounts	4	0	4
Total	46	32	79^a^
*Note*. ^a^One Sinhala language newspaper article had both an in-depth account of a case of self-harm (feature article) as well as a section providing advice about suicide (commentary).			

Photographs or drawings were included in 26% of articles, especially in the short-story-styled feature articles in the English-language *Ceylon Today*. Here, large colorful drawings of means used for self-harm/suicide were presented alongside the text. For instance, an article headlined "A Love That Took the Life of a Soldier" included a drawing of a soldier holding a gun to his head (ENG1). These articles were highly evocative, in a style reminiscent of fiction. While they portrayed a rural, poor individual in a difficult situation, the newspaper's audience would likely be located in urban areas. Other articles included photographs of the deceased and/or the location of the self-harm. In two of the Sinhala articles, a photograph of a police-person who had played a role in the case was included.

### Social Actors' Relations and Identities

A reoccurring theme was provisions of personality traits of the individuals who self-harmed. Evaluative adjectives and verbs described the individuals before their self-harm. Often this included positive evaluations:

Nipuni's mother has said that she was a really good daughter-in-law. (SIN39)

Tharindu, as an employer was a man of nobility and gentleness. He paid the salaries of his employees on time. (ENG2)

These articles memorialized the victim while expressing disbelief about the situation. Although evoking feelings of empathy toward the individual, these articles also questioned how self-harm could happen to individuals who played their social role appropriately. For example, one article stated that:

He is a very social, friendly and helpful person and he did not have a reason to commit [sic] suicide. (SIN17)

In other cases, the individuals' character was portrayed in negative terms, evoking feelings of disdain:

Their [individuals who self-harm] personality is weak. (SIN28)

She has never undergone any hardships. She does not even know what suffering or sadness mean because she led a luxurious life thanks to her father's money (…). (SIN7)

In these quotes, stigma surrounding individuals who self-harm was evident. Not only was their personality questioned, but also their reasons to self-harm. Several articles commented on the individual's children. For instance, a husband reflected that his wife had no reason to self-harm because of her love for their children. Another article highlighted how a woman should not be blamed for her self-harm although she had children:

She can't be viewed as a mother who has no love toward her children, but one that loved them more than her life and when she had failed to give them a contented life. The ignorant woman thought by ending her life [she] would end her troubles. (ENG4)

Here, the woman's behavior was excused by highlighting her ignorance while communicating how it was linked to a lack of knowledge and a paternalistic attitude of condescension. A woman's love for her children thus appeared to play a part in how her self-harm was perceived. 

### Relational Breakdown Preceding Self-Harm

A conflict between close relations was at the center of most self-harm/suicide cases presented. Suicidal individuals were often defined through their relationship with others and their actions, for example, a daughter's affair or a wife's disobedience. In some instances, the others in a relationship were portrayed as having no influence over the suicidal individual's actions:

Samadhi, on the other hand, could do nothing for her husband as she had already helped him in many ways. She, as usual, engaged in household chores. (ENG2)

In other instances, the relatives were blamed for the self-harm, thereby placing responsibility for the event with someone else:

His wife showed little relaxed attitude over some affairs of life some of which were of apparent contrast to her husband's. She cannot be called a disobedient wife, but had a habit of standing firm on her decision regardless of her husband's stand on issues. (ENG5)

In this man's instance, his suicide was explained to be rooted in a conflict over his wife's actions – she did not follow his advice in avoiding involvement in political matters. 

### Practicing an Appropriate Gendered Behavior

Often the personality traits of the individual who self-harmed and their relatives were linked to gender. Men were typically described as hardworking:

He worked hard (…) and was known in the area as a man. (ENG5)

An in-depth article describing the heartache and suicide of a soldier highlighted how:

Shehan burst into tears in secret to his fellow soldiers. He knew that if anybody would see him cry, it would be a grave damage to his bravery as a soldier who should possess a heart which can bear any agony. (ENG1)

These newspaper articles communicated a masculine behavior, where men were supposed to be self-controlled and hard-working. Women, on the other hand, were often described as disobedient and inactive:

His wife displayed her idleness. (ENG5)

In other instances, articles highlighted how women were able to bear up under problems:

She tolerated everything because of her daughters (SIN28)

An evaluation of the individual's behavior, therefore, played a pivotal role in portraying the cases of self-harm and suicide.

### Intertextuality

Six episodes of self-harm/suicide were portrayed in several newspaper articles. Often the case was related to political issues, specifically the presidential election. One article depicted a man who seemingly self-harmed owing to his wife's political engagement. Two running stories portrayed the defeat of the sitting president. One reported how a man self-harmed as a result of distress over the election results, following his story through several articles (Sinhala *n* = 3, English *n* = 3) from the act of self-harm, hospitalization, and death. Another article featured how an astrologer had "vowed to carry out an act of self-immolation" if the sitting president was not re-elected (ENG30, ENG31). In both cases, self-harm/suicide seemed to demonstrate devotion to the politician. 

Migration was another reoccurring theme mentioned in 10 of the 29 feature articles. Two cases were prominent, reporting how women had migrated abroad for work without family consent, mysteriously died, leaving relatives and newspapers speculating whether it was due to homicide or suicide. In these articles, difficult circumstances encountered by migrating women were highlighted. 

### What Is Not Said

Especially in crime reports and feature articles, information about the complexities of self-harm was missing. Instead, a mono-causal rationale for self-harm was provided:

The elder brother committed [sic] suicide when his younger brother asked him to marry the proposed girl. (SIN17)

Initial investigations revealed that the deterioration of his mental health due to his illness triggered the incidence. (ENG16)

A person has committed [sic] suicide for being afraid to go to court. (SIN30)

Except for two commentaries by health professionals focusing on the complexities of self-harm, a single, unsubstantiated cause was communicated in the articles. Only three of the included articles mentioned alcohol as a factor. None provided information about help-seeking. 

## Discussion 

To the best of our knowledge, this is the first in-depth exploration of Sri Lankan newspaper articles' portrayal of self-harm and suicide. The analysis found four relevant aspects of reporting in the selected articles:

• Individuals who self-harmed were judged according to their own or a close relations' behavior.
• Behavior was often linked to the individual's gender.
• Explanations for cases of self-harm were one-sided.
• Suicide prevention narratives were absent.

### Stepping Outside Acceptable Behavior

In the selected newspaper articles, reoccurring themes were individual behavior and relational breakdown. While some articles expressed judgment toward the individual's inappropriate behavior, others communicated shock when the individual had stayed within the boundaries of acceptable behavior, questioning how someone who played their social role appropriately would self-harm. Such norms stem from a social structure where restraint is valued and expression of emotions suppressed (Obeyesekere, 1984; Spencer, 1990a). There was also a gendered component. Traditionally, Sri Lankan men are expected to be self-controlled, guardians of women's behavior, and main breadwinners (Obeyesekere, 1984; Spencer, 1990a). Women are supposed to communicate shyness and be self-sacrificing, looking after their families before themselves (Obeyesekere, 1984; Spencer, 1990b). Women's submissiveness and love, especially to their children, was a perpetual theme in the articles. In existing literature, submissiveness has been highlighted as an inherent part of the ideal Sinhala woman's behavior (Spencer, 1990b; Widger, 2012a). In a qualitative study, Sørensen et al. (2017) found that women took on a submissive role in relation to their spouses to conform to norms of female respectability. This included being caring mothers and wives who silently took it on themselves to preserve family respectability. As is also portrayed in the newspaper articles of the current study, self-harm occurred when women stepped outside such behavior (Sørensen et al., 2017).

In addition to individual behavior, relationship breakdown was a reoccurring explanation for self-harm in the included articles. This has previously been found in Sri Lankan self-harm literature where several studies have highlighted how a purpose of self-harm is to communicate something that cannot be said in words, typically to close relations (Marecek, 1998; Sørensen et al., 2017). The importance of kinship in the context of self-harm in Sri Lanka was, for example, highlighted in an ethnographic study (Widger, 2012b) and a qualitative study of self-harm in rural Sri Lanka showed that the majority of self-harm cases were initiated by a partner-conflict (Sørensen et al., 2017). 

### Reporting the Complexities of Self-Harm

The international literature on suicide strongly emphasize how self-harm is rarely the result of a single factor (World Health Organization, 2017). Studies from Sri Lanka have found that deprivation and alcohol misuse were associated with risk of self-harm (Knipe et al., 2018) and that individuals who self-harmed were struggling with a number of life stressors, such as domestic violence, financial strain, and difficulties in living up to gendered expectations of the marriage (Sørensen et al., 2017). Although newspapers could be an outlet for challenging a simple explanation of self-harm, this was generally not the case in the selected articles. 

The newspaper-portrayal of self-harm likely influenced public perceptions of self-harm by over- and under-emphasizing certain aspects. An issue rarely mentioned in the articles was alcohol consumption, which is similar to a content analysis of newspapers in India where few articles mentioned the link between alcohol and suicide (Armstrong et al., 2018). However, it is in contrast to Sri Lankan research where self-harm has continuously been linked to alcohol intake (Pearson et al., 2014). For instance, a study of nonfatal self-poisoning found hazardous drinking or alcohol use disorders in more than one third of 419 Sri Lankan men (Rajapakse, Griffiths, Christensen, & Cotton, 2014). Although women typically do not consume alcohol in Sri Lanka (Wilsnack, Wilsnack, Kristjanson, Vogeltanz-Holm, & Gmel, 2009), it is associated with their self-harm more indirectly, for example, through the harmful effects of a husband's alcohol consumption (Konradsen, Hoek, & Peiris, 2006; Sørensen et al., 2017). 

Migration was continuously brought up in relation to self-harm. Presenting an opportunity for income generation (Sri Lanka Bureau of Foreign Employment, 2015), women in particular often choose to migrate internationally to be employed as domestic workers (Asian Development Bank, 2015). In the articles focusing on migration, appropriate behavior was a reoccurring theme. Female migration represents ultimate submissiveness where women give up everything to financially care for their families. However, they also break with norms of appropriate behavior. A recent Sri Lankan cohort study found that the risk of self-harm was elevated in households with a female migrant (Knipe et al., 2019). Especially the absence of migrating mothers have, in Sri Lanka and internationally, been viewed to be "abnormal" owing to its impact on the well-being of the left-behind children (Gamburd, 2008; Parrenas, 2005). The articles positioned the migrating women as trivial for "buying" the notion about greener pastures abroad, while educating the audience about the potential risks of migration.

### Absence of a Suicide Prevention Narrative

A striking finding was that the majority of articles included no suicide prevention narrative, consistent with other research from the region (Armstrong et al., 2018; Arafat, Khan, Niederkrotenthaler, Ueda, & Armstrong, 2020). Newspapers might choose the angle of individual behavior and relationship breakdown as a reason for self-harm since this likely resonates with readers in this setting. However, this places substantial pressure on individuals and relatives, while excluding other possible explanations for self-harm including mental illness, harmful alcohol use, or structural issues. Help-seeking was not included in the newspaper articles, although highlighted in international guidelines as imperative in suicide prevention (World Health Organization, 2017). One explanation could be that suicide is rarely linked to severe mental illnesses in Sri Lanka (Pearson et al., 2014) and that newspapers reflect this. Furthermore, mental health support is limited in Sri Lanka, although a few counseling services and hotlines do exist (CCC Foundation, 2009; Sri Lanka Sumithrayo, 2013). Sri Lankan newspapers could play an important part in creating awareness about these available services.

### Implications

This study illustrates how a public discourse on self-harm is reflected in newspaper reporting. When self-harm is simplified and stereotyped, there is a danger for imitation and that individuals who self-harm feel stigmatized, while leaving larger structural issues ignored. A next step is to engage media professionals in a dialogue about sensible reporting, which may result in guidelines sensitive to the Sri Lankan context, as exemplified in other parts of the world (Pirkis, Blood, Beautrais, Burgess, & Skehan, 2006). 

## Limitations 

Although inspired by CDA, we did not conduct a full discourse analysis (Carvalho, 2008). We analyzed the presentation of a journalistic product and were not concerned with whether the accounts represented reality. The months of newspaper article screening took place during 2014–2015, and the media situation might have slightly changed since then. Only articles from printed newspapers were included, although a move to online and social media would likely have been a consistent trend since the identification of articles for this study. Considering this media development, the analysis of newspaper articles from relevant outlets would have to be undertaken again within a few years. Further, the selected months led up to the presidential election, which might have influenced the content. An analysis of how self-harm/suicide is portrayed on social media and in television would be relevant. The Sinhala-language newspaper articles were thoroughly translated into English; however, the translation might not have fully captured certain nuances. Only English- and Sinhala-language newspapers were included although additional understanding would have been present in Tamil newspapers.

## Conclusion 

This study explored the portrayal of self-harm and suicide in Sri Lankan newspapers. Certain aspects were over- and under-emphasized. Cases were portrayed simplistically and individuals who self-harmed were judged according to their own or a close relation's behavior. The newspapers contributed with a discourse about self-harm that lacked visibility about the complexities of self-harm. There was a striking absence of a suicide prevention narrative. The findings are vital in informing future guideline development and communication strategies with media professionals. 
